# The prevalence of antibodies to *Toxoplasma gondii* in sheep in the Western Cape, South Africa

**DOI:** 10.4102/ojvr.v82i1.993

**Published:** 2015-11-03

**Authors:** Kenneth Hammond-Aryee, Lesley S. van Helden, Paul D. van Helden

**Affiliations:** 1 Division of Molecular Biology and Human Genetics, SA MRC Centre for Tuberculosis Research, DST/NRF Centre of Excellence for Biomedical and Tuberculosis Research, Stellenbosch University, South Africa; 2 Veterinary Services, Western Cape Government Agriculture, South Africa

## Abstract

The seroprevalence of *Toxoplasma gondii* antibodies in a sample of 292 merino sheep farmed in a semi-intensive manner in the Overberg region of the Western Cape, South Africa, was investigated. Antibody seroprevalence was determined by enzyme-linked immunosorbent assay. Of the total sample, 23 sheep tested positive for *T. gondii* antibodies (8%; 95% CI: 4.7688–10.9846). There was no statistically significant relationship between seroprevalence and age of the sheep. The highest seroprevalence was found in sheep between 28 and 40 months old; a total of 19 sheep were seropositive by 40 months. No seropositive sheep were found in the age group between 16 and 28 months. The seroprevalence reported in this study is higher than what has previously been reported for the Western Cape (6%) and across South Africa on average (4.7%). As sheep farming is economically significant in South Africa, the presence of *T. gondii* amongst sheep may pose a production threat to the small-stock industry as well as to public health and food security. We therefore recommend further surveillance to identify high-risk animal populations so that local control measures can be put in place.

## Introduction

*Toxoplasma gondii* is an apicomplexan, obligate intracellular protozoan parasite of global importance. *T. gondii* infection causes the disease toxoplasmosis in humans and animals and its antibodies are known to be present in about a third of the global human population, although local and regional prevalences vary widely. *Toxoplasma gondii* is very successful as a pathogen owing to its ability to infect almost all mammals and birds (Dubey 2002). Toxoplasmosis is found worldwide, but is more common at lower altitudes and in warm and humid climates.

Members of the family Felidae are the only known definitive hosts for *T. gondii*. Cats can become infected by feeding on prey already infected with dormant *T. gondii* cysts or tachyzoites and also by drinking oocyst-contaminated water. Infected cats are known to shed infective oocysts in their faeces 5–12 days post ingestion of oocysts (Al Kappany *et al*. 2010; Dubey 1995; Elmore *et al*. 2010), thereby contaminating the environment and posing a risk of transmission to other species.

Toxoplasmosis causes substantial economic losses in sheep farming globally and was first described in ovines in 1954 (Buxton *et al*. 2007; Jones & Dubey 2012). Primary *T. gondii* infections in livestock, in particular sheep and goats, pose a health risk to these animals, as infection is known to cause abortions, stillbirths and neonatal mortalities. In the United Kingdom, for example, ovine toxoplasmosis causes up to 2% of foetal losses per annum (Buxton *et al*. 2007; Innes *et al*. 2009).

In humans and animals, *T. gondii* infections are acquired post natally by the ingestion of tissue cysts in partially cooked meat, infective oocysts in food or water contaminated with infected felid faeces, or handling of tissues of animals infected with tissue cysts. Infection can also occur by vertical transmission from mother to foetus in humans, sheep, goats and small rodents (Hill & Dubey 2002; Jones & Dubey 2012; Smith 1993). In humans, infection can also occur via blood transfusions and organ transplantation, although this is rare. Infection has been known to occur via inhalation of aerosols containing infective oocysts or from contact with contaminated soils in both humans and animals.

In sheep, *T. gondii* infection is mainly acquired post natally, as congenital infections usually lead to abortions. Rarely, congenitally affected lambs are born, which can be a possible route for infection in humans (Dubey & Welcome 1988; Williams *et al*. 2005).

There are various risk factors reported to be associated with *T. gondii* infection in sheep. Seroprevalence in sheep is known to increase with age and is therefore higher in ewes or rams than in lambs (Dubey 2009). Other risk factors for *T. gondii* infection in sheep include the presence of cats on farms, the nature of farming and management practices (commercial vs non-commercial; intensive, semi–intensive, free range or open), climatic conditions and geographic location, presence of surface drinking water sources and size of the farm (Abu Samra *et al*. 2007; Andrade *et al*. 2013; Mainar *et al*. 1996). Although infected ewes do not always show symptoms of clinical toxoplasmosis, *T. gondii* infection has been noted as an important cause of ovine abortions in the United States and Europe (Dubey 2009), with seroprevalence in sheep ranging from 20.8% (Huffman *et al*. 1981) to 73.8% (Dubey & Welcome 1988) in the United States. It has also been suggested that some breeds of sheep may be more susceptible to *T. gondii* infection than others (Dubey & Welcome 1988; Williams *et al*. 2005).

In South Africa, seroprevalence studies have been reported in human populations (both asymptomatic and HIV and/or AIDS cohorts) as well as in animal populations, as reviewed by Hammond-Aryee, Esser and Van Helden (2014a). However, these studies are limited and few of them are from the post-HIV era. There have been even fewer studies in animal populations and only one focused on sheep, reporting seroprevalence as 4.3% via enzyme-linked immunosorbent assay (ELISA) and 5.6% via indirect fluorescent antibody (IFA) tests (Abu Samra *et al*. 2007). In that study, the Western Cape was also mentioned as the province with the highest consumption of mutton in South Africa.

The ingestion of partially cooked, undercooked or raw meat has been documented as a significant mode of *T. gondii* infection worldwide. Studies in Europe have shown that ingestion of undercooked lamb was a risk factor in the acquisition of *T. gondii* infection in a cohort of pregnant women (Cook *et al*. 2000). In a US study, 50% of a cohort of 131 women who had vertically transmitted *T. gondii* to their infants recalled having eaten raw or uncooked mutton sometime during their pregnancies (Boyer *et al*. 2005). As *T. gondii* can be transmitted to humans by the ingestion of mutton or lamb, sheep may have an important role in the epidemiology of toxoplasmosis. Investigation into the presence or absence of *T. gondii* antibodies in a population of sheep will provide significant insight into the risk of toxoplasmosis in a particular ecosystem.

The current study focused on the seroprevalence of *T. gondii* antibodies in a flock of sheep in South Africa. It contributes to the knowledge about this important pathogen and the role of animals in the epidemiology of toxoplasmosis.

## Materials and methods

### Study area and climate

Sheep were sampled from a farming area in Bredasdorp in the Overberg region of the Western Cape, South Africa. The area has a Mediterranean climate and receives about 350 mm of rain per year, most of which is in winter. December is usually associated with the lowest rainfall (< 20 mm), whereas August is associated with the highest rainfall (40 mm – 50 mm). The average midday temperatures are between 17.5 °C in winter and 26.2 °C in summer. Livestock farmed in the region include mostly sheep and cattle.

**FIGURE 1:**
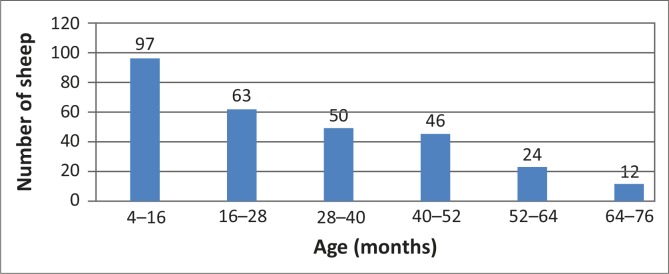
Age distribution of study sample.

The sample consisted of 292 merino sheep (4 rams and 288 ewes) selected randomly from a flock of approximately 1000–1500. The sheep were farmed in a semi-intensive manner, grazing planted pastures and crop stubble. The rams were between 16 and 64 months old, whilst the ewes were between 4 and 76 months old, with the modal age group being between 4 and 16 months (Figure 1).

### Cat presence on the farm

At the time of sampling, there were 10 domestic cats living on the farm.

### Blood collection

In May 2014, blood samples were collected from the 292 sheep via venipuncture. Blood was collected into anticoagulant-containing tubes and immediately put on ice, after which it was transported to our laboratories and centrifuged at 3500 g for 5 min to isolate the plasma. The plasma was then stored at −80 °C for further analysis.

### Serological investigations

An ELISA was used for the detection of IgG antibodies to *T. gondii* in sheep plasma samples. The ELISA test was performed via a commercially available enzyme immunoassay kit (IDEXX Toxotest Ab, IDEXX Laboratories, Switzerland) according to the manufacturer's instructions.

Briefly, thawed sheep plasma samples and controls were diluted at a ratio of 1:400 in wash buffer to prevent non-specific reactions. The diluted plasma samples were then dispensed into microtitre plates precoated with inactivated *T. gondii* antigen and mixed by gentle shaking.

The microplate was covered with an adhesive plate cover and incubated at 37 °C for 60 min. Each well was then washed three times with approximately 300 mL buffer in each wash. All residual wash solution was removed by tapping the microplate gently onto an absorbent material. Bound antigen–antibody complexes were then conjugated with 100 μL peroxidase-labelled anti-ruminant IgG conjugate. The plate was covered and incubated at 37 °C for 60 min. The described wash step was repeated to eliminate any residual unbound complexes. Enzyme-bound complexes were visualised by adding 100 μL enzyme substrate to the wells and incubating the plate at 26 °C for 15 min. The enzyme–substrate reaction was stopped by adding 100 μL stop solution to each well of the microplate. The absorbance was read on a photometer at a wavelength of 450 nm.

Positive and negative controls provided in the kit were included on each microplate per batch of test samples.

The optical densities of the positive control (*PCx*) and the samples (*SampleA*_450_) were corrected by subtracting the optical density of the negative control (*NCx*). The samples were analysed relative to the positive and negative controls:

$$S/P(\%)=100\times \frac{SampleA_{450}-NCx}{PCx-NCx}$$ [Eqn 1]

S/P is a ratio of the cosrrected optical density of the sample to the corrected optical density of the positive control for each sample.

### Statistical analysis

A Pearson chi square test was performed using SAS/STAT statistical analysis software (version 9.3). Correlations between the serological status of sheep and their age were investigated. Differences were deemed statistically significant if *P* ≤ 0.05.

## Ethical considerations

Ethical clearance for this study was obtained from the Animal Health Research Ethics Committee of the Western Cape Department of Agriculture (ethical clearance certificate G13/89).

## Results

Of the total sample, 23 sheep (8%; 95% CI: 4.7688–10.9846) tested positive for *T. gondii* antibodies.

**FIGURE 2:**
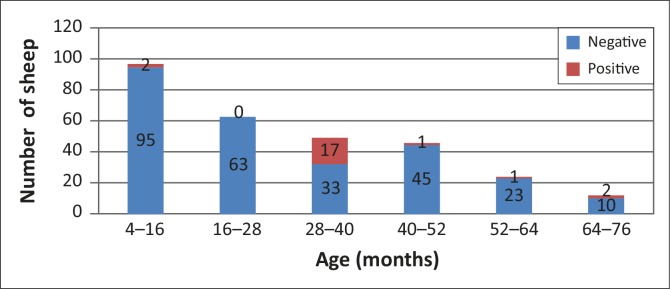
Seroprevalence of *Toxoplasma gondii* antibodies in a sample of sheep, organised according to age.

Figure 2 shows that distinct *T. gondii* antibody seroprevalence patterns were evident in the different age categories shown in Figure 1.

The highest seroprevalence (34%) was observed in animals between 28 and 40 months old, followed by a seroprevalence of 16.7% in the 64–76-month-old group. The seroprevalence in the 52–64-month-old group was 4.2%, whereas a seroprevalence of 2.1% was seen in both the 4–16-month-old group and the 40–52-month-old group. No seropositive sheep were observed in the 16–28-month-old group.

## Discussion

Infectious diseases, such as toxoplasmosis, have a substantial impact on animal productivity in sheep-farming regions of the world and some may remain undetected in flocks for prolonged periods, leading to unforeseen and unexplained abortions, foetal or newborn deaths and infertility. In some cases, these diseases lead to persistent or recurring infection in herds, resulting in poor reproductive output in the long term. Reproductive losses resulting from these diseases are a threat to the long-term economic viability of such flocks.

*Toxoplasma gondii* antibodies have been detected in naturally exposed sheep flocks worldwide. Reported seroprevalence varies from as low as 3% in Pakistan (Zaki 1995) to as high as 68% (Deconinck *et al*. 1996) in the Ivory Coast and 95.7% in Turkey (Mor & Arslan 2007).

*Toxoplasma gondii* seroprevalence in small ruminants has been reported from some parts of Africa. Hove, Lind and Mukaratirwa (2005) reported a seroprevalence of 67.9% in a population of sheep and goats from different parts of Zimbabwe. They also reported an eightfold difference in seroprevalence between sheep from a large commercial farm (10%) and sheep reared under a communal grazing system (80%), reporting the presence of many domestic cats and a high household density as potential risk factors for *T. gondii* infection. In Botswana, seroprevalences of between 10% (Binta *et al*. 1998) and 30% (Sharma *et al*. 2003) have been reported in a population of goats. In Ghana, Van der Puije *et al*. (2000) reported a seroprevalence of 33.2% in sheep and 26.8% in goats, with a higher seroprevalence reported in female animals (35.8%) than in male animals (21.1%). Significant differences in seroprevalence were found between breeds, age groups and ecological zones from which the animals were sampled. In Uganda, Bisson *et al*. (2000) reported a seroprevalence of 31% in a population of goats, with significantly higher seroprevalence in goats from urban areas than from rural areas. A strong positive correlation was demonstrated between age and seroprevalence.

In South Africa, Abu Samra *et al*. (2007) reported a seroprevalence of 5.6% using an IFA test and 4.3% according to an ELISA. The mean seroprevalence in the Western Cape, as determined by ELISA, was 6%. The authors also reported a significantly higher seroprevalence in sheep from commercial farms than in sheep from rural or informal-sector farms. A significant correlation was found between seroprevalence and the average minimum temperature. Sheep that were managed in an extensive manner had a significantly lower seroprevalence (1.8%) than those managed in a semi-intensive or fully intensive system (5.3%).

The seroprevalence reported from the current study (8%) is higher than the mean provincial seroprevalence reported by Abu Samra *et al*. (2007). This may be a result of the farm from which the sample was selected being a commercial farm with semi-intensive management, as both of these factors have been shown to contribute to increased risk for seroprevalence.

Most seropositive sheep had acquired the infection by 4 years of age. Seroprevalence is known to increase with age, with 95% of susceptible ewes seroconverting by 6 years of age (Dubey 2009). In our study, the highest number of seropositive sheep was between 28 and 40 months old; there was no significant relationship between seroprevalence and age of the sheep. These observations may be due to the sample being a convenience sample over which the investigator had no control.

## Conclusion

Transmission of *T. gondii* to humans and non-food animals is likely to be proportional to the *T. gondii* seroprevalence within food animal populations. There is a need to actively survey at-risk populations such as feral cats, wildlife and food animals within the ecosystem to design appropriate interventions for managing and preventing disease transmission. In Africa, where poverty, poor hygiene and a high burden of HIV infection exist, *T. gondii* infection is likely to have substantial implications for the health and economic well-being of the people of this continent (Hammond-Aryee, Esser & Van Helden 2014b).

The seroprevalence reported in this study is higher than the previously reported figures for the Western Cape (6%) and overall in South Africa (4.7%) (Abu Samra *et al*. 2007). Our study was more limited than the comparative study, but our results do suggest ongoing transmission of *T. gondii* in livestock. Cats on sheep farms may be a major risk factor for transmission of *T. gondii* to livestock, as suggested by a recent study in which *T. gondii* anti-IgG antibody seroprevalence amongst feral cats was found to be 37.1% (Hammond-Aryee *et al*. in press).

In South Africa sheep are farmed not only for meat production but also for the production of wool. An infectious disease such as toxoplasmosis can therefore have both health and economic implications in the country. We recommend ongoing surveillance to identify high-risk animal populations so that local control measures can be put in place to prevent interspecies spread of the disease.
